# Developing a risk prediction model for breast cancer: a Statistical Utility to Determine Affinity of Neoplasm (SUDAN-CA Breast)

**DOI:** 10.1186/s40001-017-0277-6

**Published:** 2017-09-29

**Authors:** Alaaddin M. Salih, Dafallah M. Alam-Elhuda, Musab M. Alfaki, Adil E. Yousif, Momin M. Nouradyem

**Affiliations:** 1 Faculty of Medicine, International University of Africa, Khartoum, Sudan; 2 National Academy of Health Sciences, Khartoum, Sudan; 3 College of Medicine and Veterinary Medicine, University of Edinburgh, Edinburgh, EH8 9YL UK; 4 Department of Community Medicine, Faculty of Medicine, University of Khartoum, Khartoum, Sudan; 5 National Ribat University and Central Police Hospitals, National Ribat University, Khartoum, Sudan; 6 Department of Statistics, College of Arts and Sciences, Qatar University, Doha, Qatar; 7 Department of OB/GYN, Ribat University and Central Police Hospitals, National Ribat University, Khartoum, Sudan

**Keywords:** Breast cancer, Prediction model, Risk assessment, SUDAN

## Abstract

**Background:**

Breast cancer risk prediction models are widely used in clinical settings. Although most of the well-known models were designed based on data collected from western population, yet they have been utilized for surveillance purposes in many limited-resource countries. Given the genetic variations in risk factors that exist between different races, we therefore aimed to develop and validate a tool for breast cancer risk assessment among Sudanese women.

**Methods:**

Using cross-sectional design, 153 subjects were eligible to participate in our study. Data were collected from the only couple of tertiary centers in Sudan. They underwent multiple logistic regression using purposeful selection method to build the model. Various adjustments were made to determine significant predictors. Overall performance, calibration and discrimination were assessed by *R* ^2^, *O*/*E* ratio and c-statistic, respectively.

**Results:**

SUDAN predictors of breast cancer were: age, menarche, family history, vegetables and fruits weekly servings, and type of cereals that traditional cuisine is made of. Both Nagelkerke *R* ^2^ (0.495) and *O*/*E* ratio (0.78) were good. c-statistic expressed the excellent discriminatory power of the model (0.864, *p* < 0.001, 95% CI 0.81–0.92).

**Conclusions:**

Our findings suggest that SUDAN provides a simple, efficient and well-calibrated tool to predict and classify women’s lifetime risks of developing breast cancer. Input from our model could be deployed to guide utilization of the more advanced screening modalities in resource-limited settings to maximize cost effectiveness. Consequently, this might improve the stage at which the diagnosis is usually made.

## Background

Breast cancer (BC) is the world’s most common malignancy with a constantly increasing incidence [1]. Risk prediction models assess either: [2] group odds of developing breast cancer over time as BCRAT (Gail) model, or individual risks of inheriting a mutant *BRCA1/2*like BRCAPRO, BOADICEA, and the Myriad II prevalence tables [3]. A practical overlap between the two objectives is present in some models. For instance, IBIS (Cuzick-Tyrer) mainly estimates the risk of breast cancer over time; furthermore, readouts of inheriting a mutant *BRCA*-*1/2* are there. BRCAPRO serves the two goals inversely. Cuzick-Tyrer represents the most accurate prediction tool [2].

Modern empirical models are used mainly in defining individual risk to develop breast cancer. Their use is limited to clinical settings since they require detailed family history and genetic analysis for some of them [4]. Applying them in population screening is not fully understood [5].

As group risk prediction models are used in anticipating breast cancer probability in a wide scale of population, they can help in offering benefits-harms weighted screening recommendations, managing patients, and improving risk reduction strategies. Women with 20–25% lifetime risk of BC are advised to undergo breast MRI according to American Cancer Society guidelines [6]. US Preventive Services Task Force guidelines recommend BRCA1/2 mutation testing for women at a high risk of CA breast [7]. FDA recommends prophylactic tamoxifen for women with 1.67% 5-years risk of BC [8].

Most of the known models represent analysis of data retrieved from American and British studies [9]. This is not inclusive of other populations’ circumstances and may contradict with its generalization over other communities that differ. Moreover, models like Gail and Cuzick-Tyrer consider findings of invasive and costly interventions as biopsies to improve the overall accuracy. Inconsistently, a proven major risk factor, breast density, that could be detected by simple, cheap and non-invasive mammographic scan is not incorporated in any model [2]. This study is trying to identify the risk factors for BC among Sudanese patients.

## Methods

### Study design and subjects

This is a multicenter, observational, retrospective, cross-sectional study conducted in breast clinics of Bashaier University Hospital (BUH) and Khartoum Center for Radiation and Isotopes (RICK), Khartoum, Sudan. Both are leading tertiary centers highly specialized in managing breast conditions among Sudanese and east African nationals. From October 2014 to September 2015, all eligible patients referred to either of the study facilities were considered. Inclusion criteria were: being a Sudanese female who had menarche, confirmed diagnosis with a standard triple assessment, cases of BC should be of primary type, and accepting participation without compensation. Uncertain diagnoses, breast metastasis from another primary focus, and comparison subjects with proliferative tumors were excluded. A total of 153 patients aged 32–74 years were enrolled for this study.

### Variables and outcome

Data were collected using structured data forms. Height and weight were measured using stadiometer of 1 mm accuracy and calibrated 0.01 kg sensitive medical weight scale, respectively. The investigated 34 defined risk factors include demographic (age, education, ethnicity, etc.); medical (past history of benign or malignant breast disease); family history of BC; reproductive (e.g., menarche, age of marriage and birth of the first live child, durations of pregnancy and lactation, and menopause); pharmacological (either usage of hormonal contraception or risk reducing like aspirin, tamoxifen, and raloxifene); nutritional (traditional food cereals, meat and animal products; vegetables and fruits, and sugar servings); and lifestyle risks like physical exercise, smoking and alcohol intake.

### Statistical analysis

Statistical analysis was performed using the Statistical Package for Social Sciences (SPSS), version 23.0 (SPSS, Chicago, IL, USA). Categorical variables were reported in numbers and percentages (*N*, %). Whereas, continuous variables were expressed as mean ± standard deviation (SD) at 95% confidence interval (CI).

Using SPSS, a dataset of the 153 participants was made. 2 × 2 contingency and frequency tables for the categorical and numerical variables were created to ensue occupancy of each cell. No empty cell was present. SUDAN CA Breast was built according to purposeful selection method. All risk factors individually underwent univariate analysis once-at-a-time using simple binary logistic regression. Only 13 with significant Wald *χ* ^2^ at *p* < 0.25 were qualified for a second phase analysis using multivariate logistic regression. Five variables with *p* < 0.05 were selected as predictors of the initial logit model. Parameter estimates of age, menarche, family history, and vegetables and fruits, and sorghum and millets were changed by 14.78, 8.88, 14.72, 7.67, 18.42, and 10.99%, respectively. Such levels exclude confoundings as none exceeded the 20% ceiling. Moreover, they changed insignificantly when variables dropped in phase 1 were subsequently added to the main effects model. All predictors fulfilled the assumptions of normality, absence of multicollinearity, and linearity between probability logit and age, menarche, and weekly servings of fruits and vegetables. Overall performance, discrimination and calibration of the final model were assessed for using *R* ^2^, c-statistic, and Hosmer–Lemeshow test, respectively.

### Ethical concerns

Ethical clearance of the Research Ethics Committee in National Academy of Health Sciences was obtained. Institutional approvals were also received prior to commencement of the research. Our study strictly followed Declaration of Helsinki. Informed consents were obtained from all participants after ensuring anonymity.

## Results

### Demographic and clinical features of participants

**Fig. 1:**
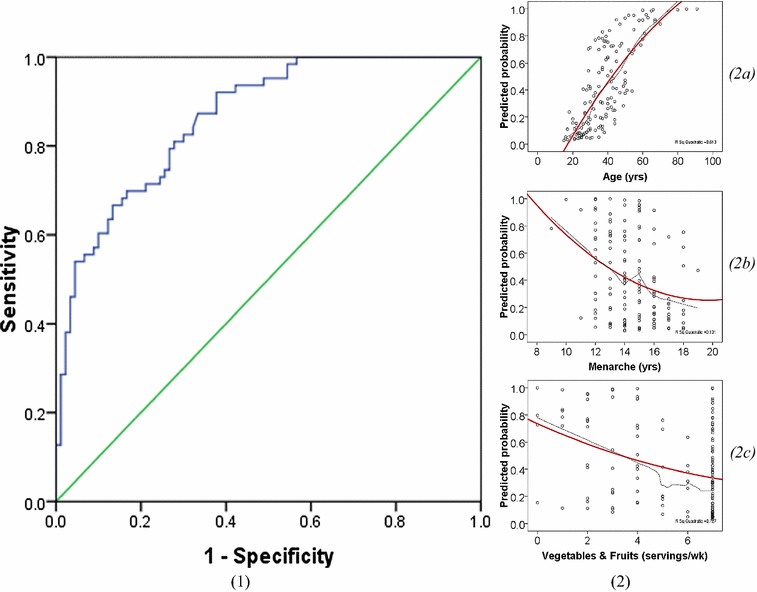
Likelihood measures of SUDAN. **1** ROC curve showing the AUC c-statistic. **2** Scatter plots of continuous data. 2a age in a polynomial relationship; 2b and 2c menarche and weekly serving of vegetables and fruits. Showing wide scattering, weak relation and negative direction of the polynomial regression line

A total of 184 patients were referred to our facilities during study period. After assessing eligibility, 153 females were incorporated in the model (Fig. 1). 63 (41.2%) subjects were diagnosed with the cancer. The median age of our sample was 37.00 (± 14.30) years. It was 46.89 (± 14.99) years for breast cancer patients and 32.64 (± 10.41) years for those who did not have the disease. Patients who were illiterate were more than those who had not BC, 34.92% compared to 17.78%. Low socioeconomic status was invariably predominant among respondents (*N* = 132; 86.27%). 120 (78.43%) of our sample came from central Sudan, whereas only 4 were from eastern states. Almost half of our African subjects had breast cancer (*N* = 26; 48.15%), compared to 37 (37.37%) of the remaining ethnicities.

Interestingly, a positive past history of breast diseases was seen in one-third of the comparison group, close to the 26.98% BC patients. Twenty (31.75%) of cancer patients had a familial pattern of the disease which existed in 12 (13.33%) of the counter group. Menarche of BC patients was significantly earlier than others mean, 14.77 ± 1.70 compared to 14.05 ± 1.92 (*p* = 0.016). Conversely, menopause of the former at 48.09 (± 5.49) years was insignificantly delayed from counterparts’ mean (*M* = 26.11 ± 2.57, *p* = 0.312). Married BC patients were 56 (88.89%), making the figure eightfold the number of singles (7, 11.11%). They experience marriage at 20.73 (± 6.98) years, one and half year prior to unaffected participants 22.14 (± 6.21, *p* = 0.245) years. Most participants with cancer have given birth to single or multiple offspring (52; 82.54%) at the age of 21.25 (± 6.28) years.

Contraception was used by only 18 (28.57%) patients, 14 of them received contraceptive pills. This was even less than those used contraception and do not gain the disease (28; 31.11%). Regular use of the prophylactic acetylsalicylic acid was used by only 4 (6.35%) and 5 (5.56%) subjects with and without malignancy. Tamoxifen and raloxifene were taken by none.

Two of the four nutritional patterns that have been studied were significant factors for breast cancer, cereals and fruits and vegetables servings. Sudanese frequently eat their traditional meals that are rich in starchy ingredients. Types of cereals, meals are made of, were investigated. Among the 23 (15.03%) subjects who consume millet, 69.57% harbor BC. Most of our samples eat vegetables and fruits throughout the week with an indiscriminate mean between healthy and ill individuals, 6.62 (± 0.958) and 6.79 (± 0.679) servings per week, respectively.

### Uni- and multivariate analysis

Table 1 contains the results of the univariate analysis of the 34 known risk factors resulted in only 13 significant at *p* < 0.25. When incorporated into a single multivariate logistic regression, the 5 with *p* < 0.05 were the predictors (Table 2).

**Table 1:** Main effects model containing significant factors (*p* < 0.25) underwent univariate analysis

Factors	Mean (± SD)/*N* (%)	*β*	S.E.	*p* value	OR	95% CI
Demographic risks						
Age (years)	38.5 (± 14.3)	0.093	0.018	0.000	1.098	1.06–1.14
Educational level						
Illiterate	38 (24.8%)	0.909	0.382	0.017	2.482	1.17–5.25
Literate	115 (75.2%)				(Reference)	
Occupational status						
Unemployed	111 (72.5%)	1.073	0.409	0.009	2.924	1.31–6.52
Employed	42 (27.5%)				(Reference)	
Ethnicity						
African	54 (35.3%)	0.442	0.343	0.197	1.556	0.80–3.05
Non-African	99 (64.7%)				(Reference)	
Family						
Family history of BC						
NA	121 (79.1%)				(Reference)	
YA	32 (20.9%)	1.106	0.412	0.007	3.023	1.35–6.77
Reproductive						
Menarche (years)	14.5 (± 1.8)	− 0.227	0.096	0.018	0.797	0.66–0.96
Marital status						
Single	33 (21.6%)				(Reference)	
Married/have married	120 (78.4%)	1.179	0.463	0.011	3.250	1.31–8.06
Birth history						
Nulliparous	43 (28.1%)				(Reference)	
Parous	110 (71.9%)	0.959	0.398	0.016	2.608	1.20–5.69
Nutritional						
Traditional food cereals (servings/week)						
Wheat	107 (69.9%)				(Reference)	
Sorghum/Milo	23 (15.0%)	1.249	0.476	0.009	3.488	
Millet	23 (15.0%)	1.634	0.499	0.001	5.126	
Vegetables & fruits (servings/week)	5.4 (± 2.2)	− 0.212	0.078	0.007	0.809	
Sleep						
Nocturnal sleeping (h)	7.19 (± 1.7)	− 0.143	0.101	0.157	0.867	0.71–1.06
Measurements						
Weight (kg)	66.0 (± 16.1)	0.014	0.010	0.188	1.014	0.99–1.03
Body mass index (kg/m^2^)	24.6 (± 5.6)	0.041	0.030	0.166	1.042	0.98–1.11

**Table 2:** Five predictors of SUDAN CA Breast with their parameter estimates

Predictors	*Β*	S.E.	*p*-value	*OR*	95% CI
Age (years)	0.098	0.021	0.000	1.103	1.057–1.150
Menarche(years)	− 0.318	0.124	0.010	0.727	0.571–0.926
Family history of BC					
NA	0.000			1 (Ref)	
YA	1.141	0.504	0.024	3.130	1.166–8.406
Vegetables & fruits (servings/week)	− 0.265	0.112	0.018	0.767	0.616–0.956
Traditional food cereals (servings/week)					
Wheat	0.000			1 (Ref)	
Sorghum/Milo	1.098	0.599	0.067	2.997	0.927–9.688
Millet	1.676	0.659	0.011	5.345	1.468–19.463

A logistic regression was performed to assess the effects of age, menarche, family history, vegetables and fruits weekly servings, and type of cereals that traditional cuisine is made of in causing BC. The logistic regression model was statistically significant (*χ* ^2^ = 70.027, *p* < 0.001). SUDAN had successfully classified 49.5% of variance existed between the two groups (Nagelkerke *R* ^2^ = 0.495). Age has a significant positive relation with breast cancer. An increase in age by 1 year increases the odds of BC by 1.103 compared to the preceding year. Conversely, menarche is inversely related to the disease with each year of delay that minimizes lifetime risks by 0.273. Positive family history of BC triples likelihood of developing malignancy during lifetime. Increasing the number of weekly servings of vegetables and fruits by a single digit reduces the risk of BC by 0.232. Classical Sudanese gastronomy is based on three main cereals. Using wheat as a reference point, subjects who depend on sorghum had a threefold increase in their odds of breast cancer. Even more, millet tops risk up by five times.

### Model performance

SUDAN was assessed in terms of overall performance, discrimination and calibration using *R* ^2^, c-statistic, and Hosmer–Lemeshow Goodness of fit (GOF) test, respectively. With a Nagelkerke *R* ^2^ of 0.495, the model is capable of justifying almost half of the variance in breast cancer. Discriminatory power measured by c-statistic of AUC equals to 0.864 (*p* < 0.001, 95% CI 0.81–0.92). Hosmer–Lemeshow GOF test showed an insignificant difference between observed and predicted probabilities making the model well calibrated (*χ* ^2^ = 7.159, 8 df, *p* = 0.520). Internal validation of the model revealed an *O*/*E* ratio of 0.78.

## Discussion

To the best of our knowledge, SUDAN is the pioneering tool to assess risk of developing BC in Sudan, Africa, and Middle East. Moreover, it is the first ever of its kind been built using purposeful selection technique. It represents a multivariate logistic regression analysis of data collected in a cross-sectional study, which specified five predictors of breast cancer: age, menarche, family history, vegetables and fruits weekly servings, and type of cereals used. The former three are shared by Gail and Cuzick-Tyrer models. Claus, BRCAPRO, and BOADICEA have the same triad except menarche [10]. None of our nutritional factors was a predictor in any of the previous models. However, the relationship between nutrition and BC has been established in many studies. Kamath et al. [11] stated that strict vegetarians are three times less likely to have the disease (OR 2.80, 95% CI 1.15–6.81). Another study found a significant association between starchy food, like cereals, and certain categories of BC, ER-ve subtypes [12]. By increasing weekly servings of vegetables and fruits and replacing millet and sorghum by wheat, risks get minimized in favor of primary prevention.

The two most important predisposing factors for BC are increasing age and positive family history [13]. The presence of menarche among predictors can be expressed molecularly. Association between reproductive risk factors of BC, including menarche, and ER+ tumors, has been previously described [14, 15]. Fadl Elmoula et al. [16] reported a similar prevalence of ER+ among Sudanese and western women. On the contrary, since ER+ status is much less among Asians [17–19], risk prediction model for Thai appeared without menarche. Exceptionally, as receptor status of Koreans and western women are comparable [20], four out of KoBCRAT predictors were reproductive risks.

With an OR of 3.13, familial factors appear to play a key role in causing breast cancer. Genetically, breast cancer has autosomal dominant inheritance, predominantly for BRCA1/2 genes. Elnour et al. [21] found that BRCA1/2 mutations to be common in Sudan. Awadelkarim et al. [22] reached similar conclusions; moreover, they highlighted that 28% of their patients’ germline mutations to be novel. Furthermore, 24.3% of identified point mutations studied by Biunno et al. [23] poses unknown clinical significance, and 42.4% of them are unique to Africa.

The significance of family history and consequently BRCA1/2 predisposition may be attributed to the widespread consanguineous marriages in Sudan. The nation has the highest global rate of matrimonies involving couples who are first degree cousins (45%) [24]. This was even higher in Saha et al.’s [25] study (49.5%), with additional 13% of participants married to relative husbands of further degrees.

SUDAN has a concordance index of 0.864, which is described as excellent [26]. This was much higher than the reported c-statistic of Gail Model in a study among western women (meta-analyzed c-statistic = 0.63; 95% CI 0.59–0.67) [27]. KoBCRAT has a c-statistics of 0.63 for < 50 year women and 0.65 for others [28]. This high discriminatory accuracy qualifies our model to meet screening purposes and define population at high-risk, which better redirect our limited resources [29].

SUDAN model *O*/*E* ratio of 0.78 makes it second to Cuzick-Tyrer only (0.81; 95% CI 0.62–1.08), according to Amir et al. [30]. Corresponding values for Claus, BRCAPRO, and Gail were 0.56 (95% CI 0.43–0.75), 0.49 (95% CI 0.37–0.65), and 0.48 (95% CI 0.37–0.64), respectively. Since our overall *O*/*E* ratios of less than 1.00, subjects that really had the disease were less than expected. This raises false positives; however, it is acceptable as it increases catchment and, consequently, screening purposes which represent the principle aim of our model. *O*/*E* ratio will improve with an increase in the sample size as it better assesses predictors.

### Limitations

This study has some limitations which are addressed here. Firstly, since the cross-sectional design was used, causality relationship for significant variables could not be proven. Secondly, the model offers group predictions in the form of OR. Individual relative risks (RR) could not be calculated as the study design was not longitudinal follow-up. Thirdly, although regression sample size is an issue that is not agreed upon, however, it is relatively small though fulfilling rule of ten.

## Conclusion and recommendations

Breast cancer constitutes a real problem in Sudan. Early onset, late presentation, and limited resources are the characteristics unique to our national context of BC. This triad could be better dealt with by inventing a prediction tool. SUDAN considers risk factors in women of different ages and ranks them accordingly. This makes them aware of their odds and subsequently improves early detection. Cost effectiveness will be enhanced further by recommending screening measures for high-risk individuals. Our model showed good calibration and excellent discriminatory power.

Factors influence the role of family history and the prevalent BRCA1/2 predisposition are results of the high rates of consanguineous marriage. However, further genetic studies on the penetrance of BRCA1/2 and predisposition to other mutant genes should be considered. Utilization of purposeful selection method is advisable particularly when sample size used is relatively small. The authors support previous recommendations on establishing a national registry for cancer in Sudan. This will offer a potential data source for externally validating this model and developing it further to better fit Sudanese women.

## Abbreviations

BC: breast cancer
KoBCRAT: Korean Breast Cancer Risk Assessment Tool
O/E ratios: observed/expected ratio
OR: odd ratio
RR: risk ratio
SUDAN-CA Breast: Statistical Utility to Determine Affinity of Neoplasm (SUDAN Model)
